# DNA replication initiation timing is important for maintaining genome integrity

**DOI:** 10.1128/jb.00175-25

**Published:** 2025-07-21

**Authors:** Tristan T. Reed, Abigail H. Kendal, Katherine J. Wozniak, Lyle A. Simmons

**Affiliations:** 1 Department of Molecular, Cellular, and Developmental Biology, University of Michigan 1259, Ann Arbor, Michigan, USA; The Ohio State University, Columbus, Ohio, USA

**Keywords:** CcrZ, *Bacillus subtilis*, DnaA, DNA replication, RecA

## Abstract

DNA replication is regulated by factors that promote or inhibit initiation. In *Bacillus subtilis,* YabA is a negative regulator of replication initiation, while the newly identified kinase CcrZ is a positive regulator. The consequences of under-initiation or over-initiation of replication on genome stability remain unclear. In this work, we measure the origin-to-terminus ratio as a proxy for replication initiation activity. We show that Δ*ccrZ* and several *ccrZ* alleles under-initiate DNA replication, while ablation of *yabA* or overproduction of CcrZ leads to over-initiation. We find that cells under-initiating DNA replication have few incidents of replication fork stress as determined by the low frequency formation of RecA-GFP foci compared with wild type. In contrast, cells over-initiating replication show levels of RecA-GFP foci formation analogous to cells directly challenged with DNA-damaging agents. We show that cells under-initiating and over-initiating DNA replication are both sensitive to mitomycin C, demonstrating that changes in replication initiation frequency cause an increase in sensitivity to genotoxic stress. With these results, we propose that cells under-initiating DNA replication are sensitive to DNA damage due to asynchronous DNA replication, leading to inefficient homologous recombination. In cells over-initiating replication, we propose that an increase in the number of replication forks leads to replication fork stress, which is further exacerbated by chromosomal DNA damage. Together, our study shows that DNA replication initiation frequency must be tightly controlled because changes in initiation influence replication fork fate and the capacity of cells to efficiently repair damage to their genetic material.

**IMPORTANCE:**

The regulation of DNA replication is fundamental to cell growth and cell cycle control. In eukaryotes, under-initiation or over-initiation leads to genome instability. In bacteria, it is unclear how changes in replication initiation frequency impact DNA replication status and genome integrity. We show that tight regulation of DNA replication initiation is critical for maintaining genome integrity. Cells over-initiating or under-initiating DNA replication are sensitive to DNA damage. Furthermore, cells over-initiating DNA replication experience replication fork stress at levels that phenocopy those observed in cells challenged with DNA damage from mitomycin C. Our results establish the critical importance of properly regulating DNA replication initiation frequency because an imbalance in initiation results in replication fork perturbations, deficiencies in DNA repair, and genome instability.

## INTRODUCTION

All organisms are required to replicate their genomic DNA at the proper time during growth or the appropriate stage of the cell cycle. DNA replication initiation is a carefully regulated process that ensures replication occurs at the proper time. DNA replication initiation in bacteria and eukaryotes is mediated by initiation proteins that are part of a superfamily of ATPases associated with diverse cellular activities (AAA+) (1–5). In bacteria, DnaA is a highly conserved AAA+ superfamily protein required for replication initiation from the origin, *oriC* (3, 6). DnaA binds ATP and then the DnaA boxes located within *oriC* (3, 6). Once bound, DnaA unwinds the AT-rich region of *oriC* and associates with helicase loader proteins (2, 7). After the helicase is loaded, the remaining proteins assemble to allow for priming and bidirectional replication of the chromosome (2, 8).

Because DnaA represents the committed step to replication initiation, DnaA is subject to positive and negative regulation (3). In *Escherichia coli,* DnaA is negatively regulated by processes that limit its ATP binding or hydrolysis (9–11), while in the Gram-positive bacterium *Bacillus subtilis,* negative regulators tend to disrupt DnaA filament formation at *oriC* (12–14). YabA and Soj/ParA are examples of negative regulators of DNA replication in *B. subtilis* that interfere with the ability of DnaA to form a nucleoprotein filament at *oriC* (12, 13, 15–18). YabA has been shown to bind DnaA and the replication sliding clamp DnaN (14, 18). Binding of YabA to DnaA interferes with the cooperative binding of DnaA to *oriC,* inhibiting initiation (14, 18). Therefore, cells bearing a *yabA* null allele show asynchronous replication initiation and over-initiation of DNA replication because negative control of DnaA is relieved in the absence of YabA (14–16).

In *E. coli,* there are multiple examples of DnaA variants that cause hyperactive initiation of DNA replication (11, 19, 20). The most well-studied example is DnaAcos, which confers a cold-sensitive phenotype caused by over-initiation of DNA replication (20–23). Studies of over-initiation of replication in *E. coli* have shown that over-initiation can lead to enrichment of replication forks in origin-proximal DNA, suggesting that forks have slowed, stalled, or become blocked following excessive initiation events (19). In *B. subtilis,* the *dnaA1* allele (*S401F*) is defective for replication initiation at the nonpermissive temperature (16, 24). Furthermore, the *dnaA1* allele was recently shown to cause over-initiation of DNA replication at the permissive temperature (16). Combining *dnaA1* and Δ*yabA* resulted in a synthetic lethal phenotype, demonstrating that excessive over-initiation in *B. subtilis* results in cell death (16). In eukaryotes, over-initiation is caused by overexpression of oncogenes, cyclin E and Ras, resulting in genome instability through chromosomal breakage at fragile sites and collisions between replication forks and transcription complexes (25–27). Furthermore, in eukaryotes, under-replication also leads to genome instability, including DNA breakage at fragile sites (28–30). In the case of eukaryotes, DNA replication initiation usually occurs once per cell cycle at each origin. For bacteria, including *E. coli* and *B. subtilis,* multiple initiation events occur during each cell cycle when cells are grown in rich medium, allowing for an increase in growth rate as new cells inherit chromosomes with forks having already initiated at the origin before cell division occurs ([31], for review, see references 32, 33). The effects of under-initiation and over-initiation on genome integrity in bacterial cells using multi-fork replication remain unclear.

In *E. coli,* there are a number of proteins that help stimulate the initiation activity of DnaA, including IHF and Fis (for review, see reference 3). CcrZ is a recently identified kinase in *B. subtilis* that stimulates DNA replication initiation through *oriC* (34). CcrZ is conserved in the phylum Firmicutes and has been shown to be important for positively regulating initiation in *Streptococcus pneumoniae*, *Staphylococcus aureus,* and *B. subtilis* (34). In each of the organisms tested, deletion or depletion of *ccrZ* resulted in a lower *oriC/ter* ratio than wild-type (WT) cells (16, 34). Integrity of the kinase active site of CcrZ is required for function, and CcrZ has been shown to bind DnaA and the *B. subtilis* helicase loader protein DnaB (34, 35). The cognate substrate of CcrZ remains unclear. It has been shown that both ribose and 2-deoxyribose stimulate ATPase activity of CcrZ, suggesting that CcrZ phosphorylates a small signaling molecule (35). Ectopic expression of *ccrZ* from a xylose-inducible promoter has been shown to result in hyperactive initiation of DNA replication, sensitivity to DNA damage, and induction of the SOS response in a subpopulation of cells (35). Unlike the DnaAcos variant from *E. coli,* which leads to clear replication fork congestion near the origin, ectopic expression of CcrZ in *B. subtilis* increased initiation frequency but did not lead to replication fork pileup near the origin or anywhere else on the chromosome (19, 35).

We took advantage of the multiple methods to cause over- or under-initiation of replication in *B. subtilis* to determine how changes in initiation relate to genome instability. We show that replication fork integrity is correlated with the frequency of replication initiation: decreased initiation leads to fewer fork problems, while increased initiation results in fork stress consistent with cells incurring significant chromosomal DNA damage. We also show that under-initiation results in a failure to efficiently repair DNA damage, resulting in sensitivity to genotoxic stress. With our data, we suggest that under-initiation leads to DNA damage sensitivity caused by asynchronous replication initiation and inefficient homologous recombination. Together, our work shows that initiation frequency is carefully regulated to safeguard the genome from replication fork problems and allow for efficient DNA repair when genome integrity is compromised.

## RESULTS

### Overproduction of CcrZ bypasses negative regulation and leads to excessive over-initiation of DNA replication

**Fig 1:**
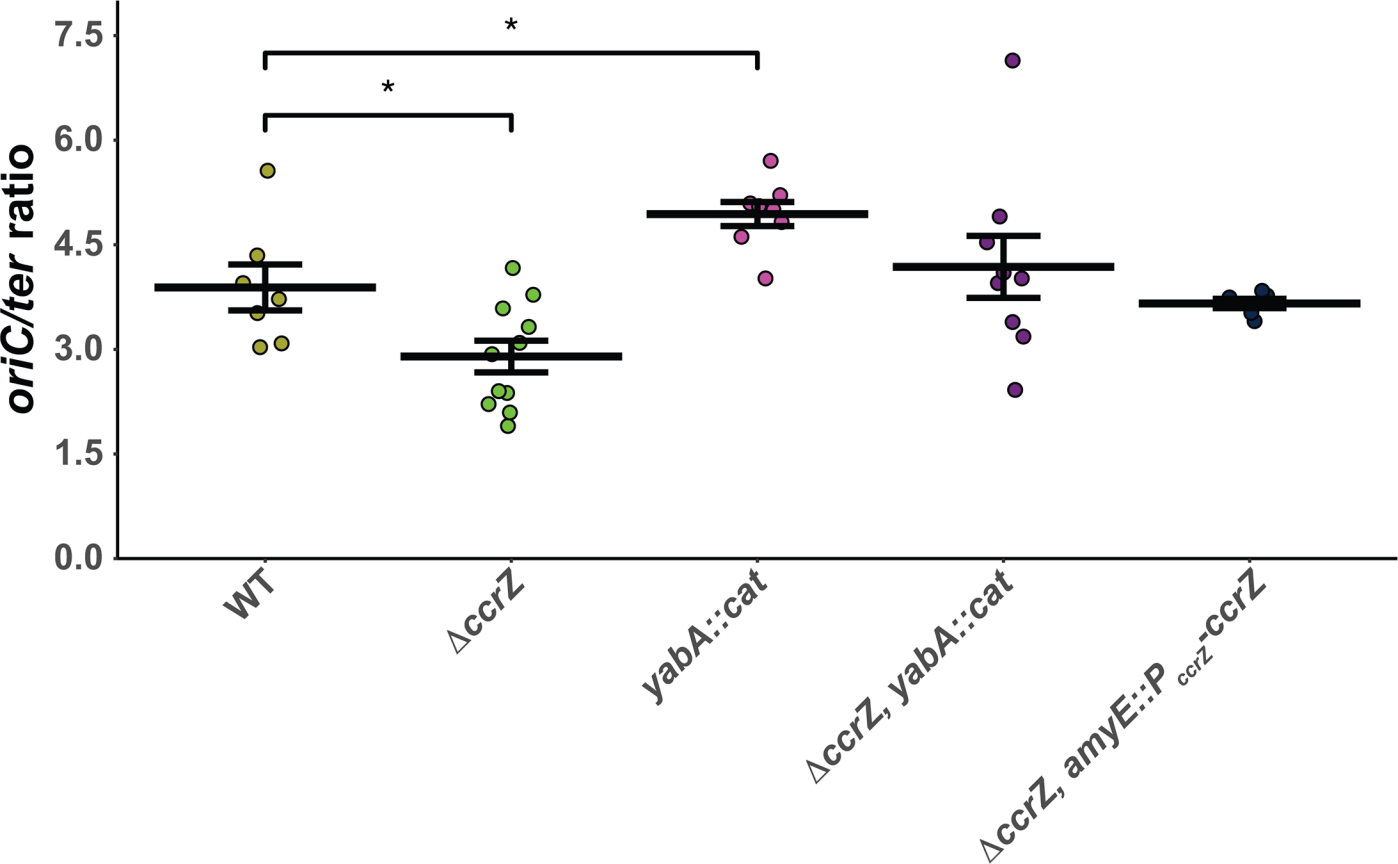
Establishing *oriC/ter* ratio of CcrZ expression under the *amyE* locus. Shown are qPCR-generated *oriC/ter* ratios for the indicated *B. subtilis* strains, grown in LB medium at 37°C, as described in Materials and Methods. Data points are plotted alongside the means represented by a horizontal bar and error bars representing the standard error of the mean for at least six independent replicates. Statistical significance of increased or decreased ratios compared to WT was assessed by two-tailed *t*-test, *P < 0.05, **P < 0.01, ***P < 0.001, and ****P < 0.0001. The data shown here are also summarized in Table 1.

The rationale for this work is to understand the effect of over- and under-initiation of DNA replication on genome integrity. Prior work from our lab showed that overexpression of *ccrZ* led to over-initiation of DNA replication as determined by whole genome resequencing (35). Work by other groups has shown that deletion of *ccrZ* results in under-initiation of DNA replication in *B. subtilis*, *S. pneumoniae,* and *S. aureus,* suggesting that the contribution of CcrZ to DNA replication initiation is conserved (16, 34). To begin, we used the same growth conditions and qPCR procedure as described previously (16) to quantify the effect of Δ*ccrZ* on DNA replication initiation by measuring the origin to terminus ratio (*oriC/ter*) (Fig. 1; Table 1). In WT cells grown in rich medium (LB) at 37°C, we show that the *oriC/ter* ratio is 3.9 ± 0.3, a result consistent with multiple published reports (16, 34, 36). Cells carrying Δ*ccrZ* under-initiate, showing an *oriC/ter* ratio of 2.9 ± 0.2, which is significantly lower than WT (*P* < 0.05), supporting previous findings (16, 34). For comparison, we also quantified the *oriC/ter* ratio in cells lacking the DNA replication initiation negative regulator *yabA* and found a ratio of 4.9 ± 0.2, a measurement significantly different from WT (*P* < 0.05). When Δ*ccrZ* was combined with the *yabA* null allele, the ratio was 4.2 ± 0.4, yielding a ratio not different from WT, supporting prior work that under- and over-initiation effects can balance to yield a WT *oriC/ter* ratio (16, 34). As a control, we ectopically expressed *ccrZ* from the *ccrZ* promoter at *amyE* in a Δ*ccrZ* background (Δ*ccrZ*, *amyE::P_ccrZ_-ccrZ*), complementing the under-initiation effect with an *oriC/ter* ratio measurement of 3.7 ± 0.06 (Fig. 1; Table 1). We also tested the effect of Δ*ccrZ* and the *yabA* null on DNA replication initiation frequency with cells grown in minimal medium and showed the same overall trend as our observations described above in rich medium (Fig. S1; Table 1).

**TABLE 1:** Summary of *oriC/ter* ratios^*a*^

Genotype	Growth medium	*oriC/ter*
WT	LB	3.9 ± 0.3
Δ*ccrZ*	LB	2.9 ± 0.2
*yabA::cat*	LB	4.9 ± 0.2
Δ*ccrZ, yabA::cat*	LB	4.2 ± 0.4
Δ*ccrZ, amyE::P_ccrZ_-ccrZ*	LB	3.7 ± 0.06
Δ*ccrZ, amyE::P_xyl_-ccrZ*	LB	10.0 ± 0.7
Δ*ccrZ, amyE::P_xyl_-ccrZ-D166A*	LB	4.1 ± 0.4
Δ*ccrZ, amyE::P_ccrZ_-ccrZ-D166A*	LB	2.4 ± 0.09
*ccrZ*^+^, *amyE::P_ccrZ_-ccrZ-D166A*	LB	3.1 + 0.4
Δ*ccrZ, amyE::P_ccrZ_-ccrZ-F47A*	LB	2.5 ± 0.2
Δ*ccrZ, amyE::P_ccrZ_-ccrZ-S103A*	LB	3.7 ± 0.3
Δ*ccrZ, amyE::P_ccrZ_-ccrZ-N171A*	LB	2.9 ± 0.04
Δ*ccrZ, amyE::P_ccrZ_-ccrZ-D184A*	LB	2.9 ± 0.06
WT	S7_50_+ arabinose	1.5 ± 0.04
Δ*ccrZ*	S7_50_+ arabinose	1.2 ± 0.07
*yabA::cat*	S7_50_+ arabinose	1.9 ± 0.05
Δ*ccrZ, yabA::cat*	S7_50_+ arabinose	1.7 ± 0.1

^*a*^ Cultures were grown in the indicated medium, followed by determination of the *oriC/ter* ratio as described (16). Data shown are the mean from at least six independent cultures, with the error representing the standard error of the mean. The data shown here represent the summary of data shown in graph form in Fig. 1 to 3 and Fig. S1.

**Fig 2:**
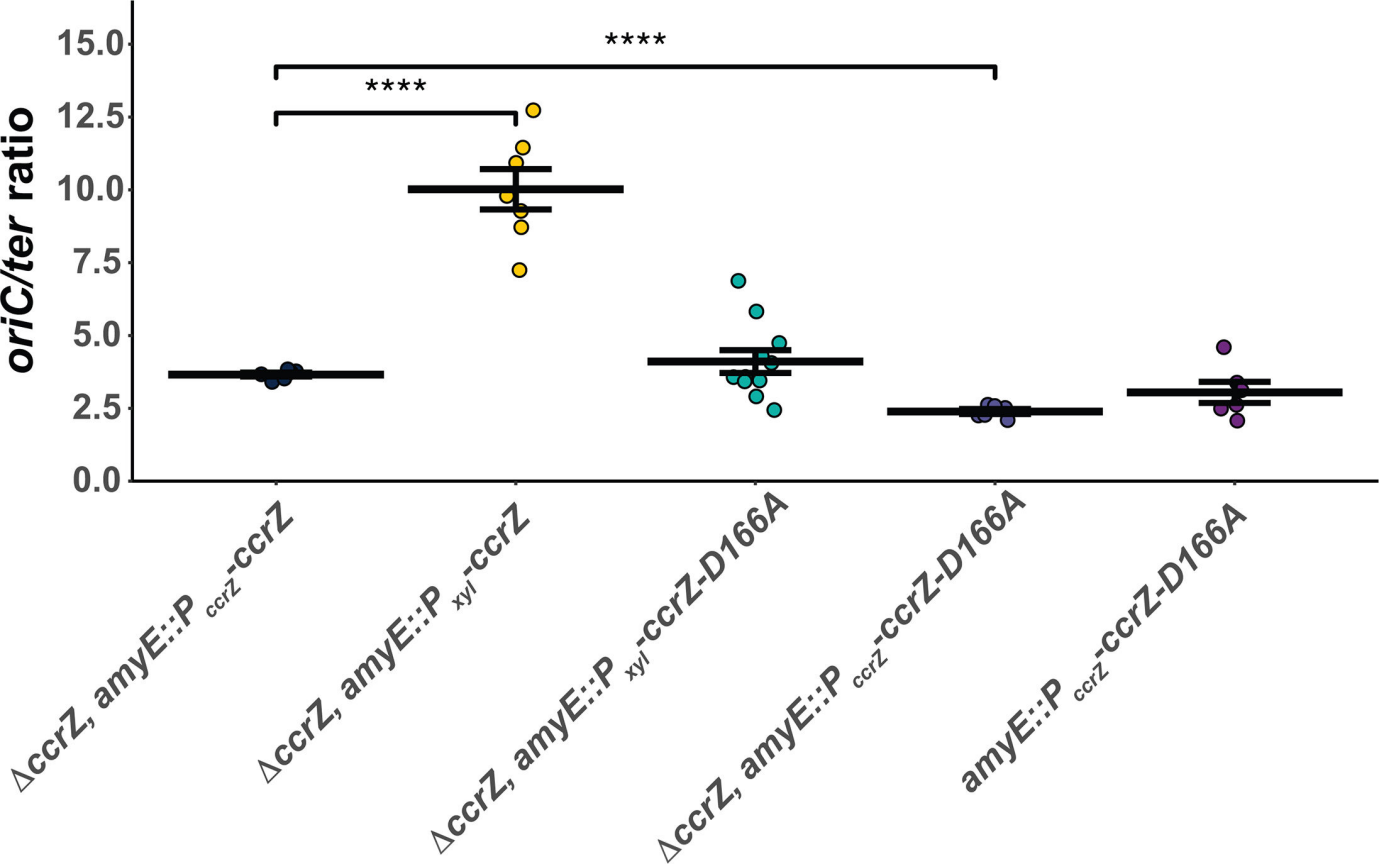
*ccrZ* and *ccrZ* mutant D166A under xylose-inducible promoters lead to increased *oriC/ter* ratios. Strains were grown in LB medium and 0.25% xylose for all strains with a xylose-inducible promoter. The *oriC/ter* ratios generated by qPCR are shown as individual data points. The crossbar represents the mean, and the error bars shown are the standard error of the mean for at least six independent replicates. Statistical significance was assessed by two-tailed *t*-test compared to Δ*ccrZ, amyE::P_ccrZ_-ccrZ.* *P < 0.05, **P < 0.01, ***P < 0.001, and ****P < 0.0001. The data shown here are also summarized in Table 1.

To determine *oriC/ter* ratios of over-initiating strains, we induced expression of *ccrZ* using a xylose-inducible promoter from an ectopic chromosomal locus (Δ*ccrZ*, *amyE::P_xyl_-ccrZ*). When *ccrZ* expression was induced, we quantified an *oriC/ter* ratio of 10.0 ± 0.7, 2.5-fold higher than WT and more than 3.4-fold greater than Δ*ccrZ* (Fig. 2; Table 1). Our qPCR results are similar to genome re-sequencing experiments showing that overproduction of CcrZ results in an elevated *oriC/ter* ratio (35). Collectively, our experiments show that overproduction of CcrZ results in a striking increase in replication initiation, a more severe initiation phenotype than our measurement of 4.9 + 0.2 for cells lacking *yabA*. We conclude that overproduction of *ccrZ* results in an excessive increase in replication initiation activity.

### *B. subtilis ccrZ* mutants under-initiate DNA replication

Based on the crystal structure of CcrZ (35), we tested several mutants for replication initiation activity *in vivo*. CcrZ(D166A) has been purified and shown to be defective for kinase activity (35). We overproduced *ccrZ*(*D166A*) in a Δ*ccrZ* background and measured an *oriC/ter* ratio of 4.1 ± 0.4, a measurement that is the same as WT but higher than Δ*ccrZ* (Fig. 2; Table 1). Since CcrZ has been shown to interact with DnaA and DnaB, we hypothesized that the initiation stimulation is caused by interactions between kinase-inactive CcrZ and DnaA or DnaB when CcrZ is overexpressed. To test this, we complemented Δ*ccrZ* with *ccrZ*(*D166A*) expressed from its own promoter from the *amyE* ectopic locus and measured a lower *oriC/ter* ratio of 2.4 ± 0.09, which is similar to Δ*ccrZ*. When *ccrZ*(*D166A*) was expressed ectopically with the WT *ccrZ* allele intact, we measured a ratio of 3.1 ± 0.4, which is lower than the WT ratio of 3.9 ± 0.3, but higher than the *D166A* ratio of 2.4 ± 0.09 (Fig. 1 and 2; Table 1). With these results, we show that CcrZ(D166A) is indeed defective in replication initiation. However, when this variant is overproduced, it is able to partially compensate for the loss of kinase activity through stimulatory interactions with DnaA and/or DnaB, resulting in an *oriC/ter* ratio close to WT levels while also conferring sensitivity to DNA damage (Fig. S2) (see Discussion).

**Fig 3:**
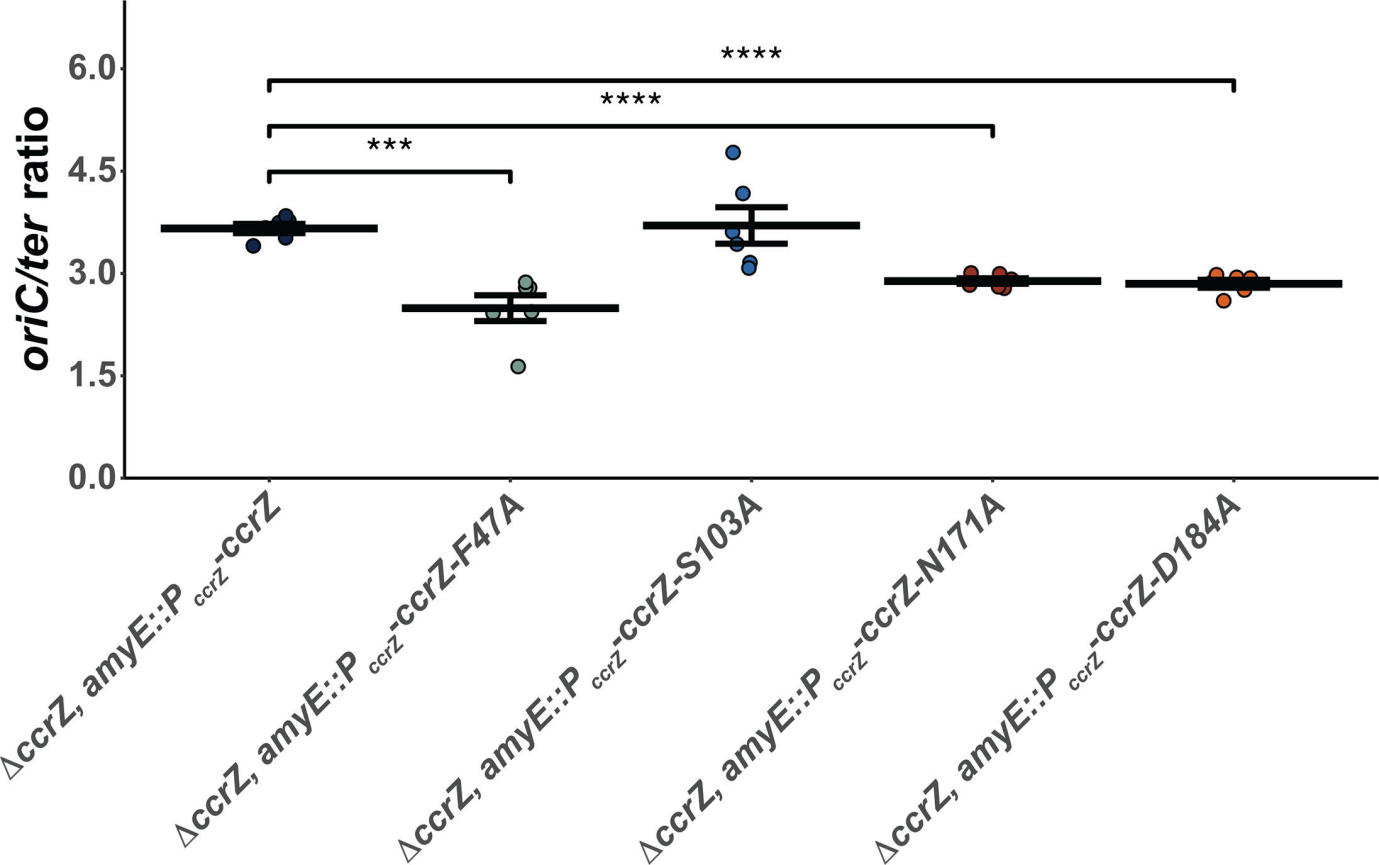
Mutations that disrupt *ccrZ* function have lower *oriC/ter* ratios. Various strains carrying *ccrZ* mutants were grown in LB media, with *oriC/ter* ratios assessed by qPCR. The individual data points are shown. The horizontal bars indicate the mean, while the error bars represent the standard error of the mean for each strain. The data shown are from six independent replicates. Statistical significance compared to Δ*ccrZ, amyE::P_ccrZ_-ccrZ* assessed by two-tailed *t*-test, *P < 0.05, **P < 0.01, ***P < 0.001, and ****P < 0.0001. The data shown here are also summarized in Table 1.

Because we found that overproduction of CcrZ(D166A) yields a WT ratio of *oriC/ter*, we tested *ccrZ F47A, S103A, N171A,* and *D184A* variants, each driven by the *ccrZ* promoter in cells with the *ccrZ* deletion allele (Fig. 3; Table 1). Residues N171 and D184 are hypothesized to coordinate Mg^2+^ based on the crystal structure (35). F47 provides a hydrophobic group at the interlobal cleft, hypothesized to maintain proper distance between the N and C terminal lobes of the enzyme (35). S103 is predicted to be important for forming an interface between the last two alpha helices on the C-terminus (35). The *ccrZ* mutants *F47A*, *N171A*, and *D184A* supported replication activity indistinguishable from Δ*ccrZ*, indicating these mutants are indeed compromised for CcrZ activity *in vivo* (Fig. 3; Table 1). We found that S103A was active in replication initiation, yielding a ratio of 3.7 ± 0.3, very close to that of WT. With our results, we suggest that F47 provides a critical role for enzyme activity in maintaining the proper distance between the N and C-terminal lobes of the enzyme, while S103A results in a WT replication initiation frequency. All defective protein variants tested here have been shown to accumulate to WT levels *in vivo* (35), indicating that the defects we observe in replication initiation are not due to low levels of expression or low accumulation of CcrZ variants in the cell.

**Fig 4:**
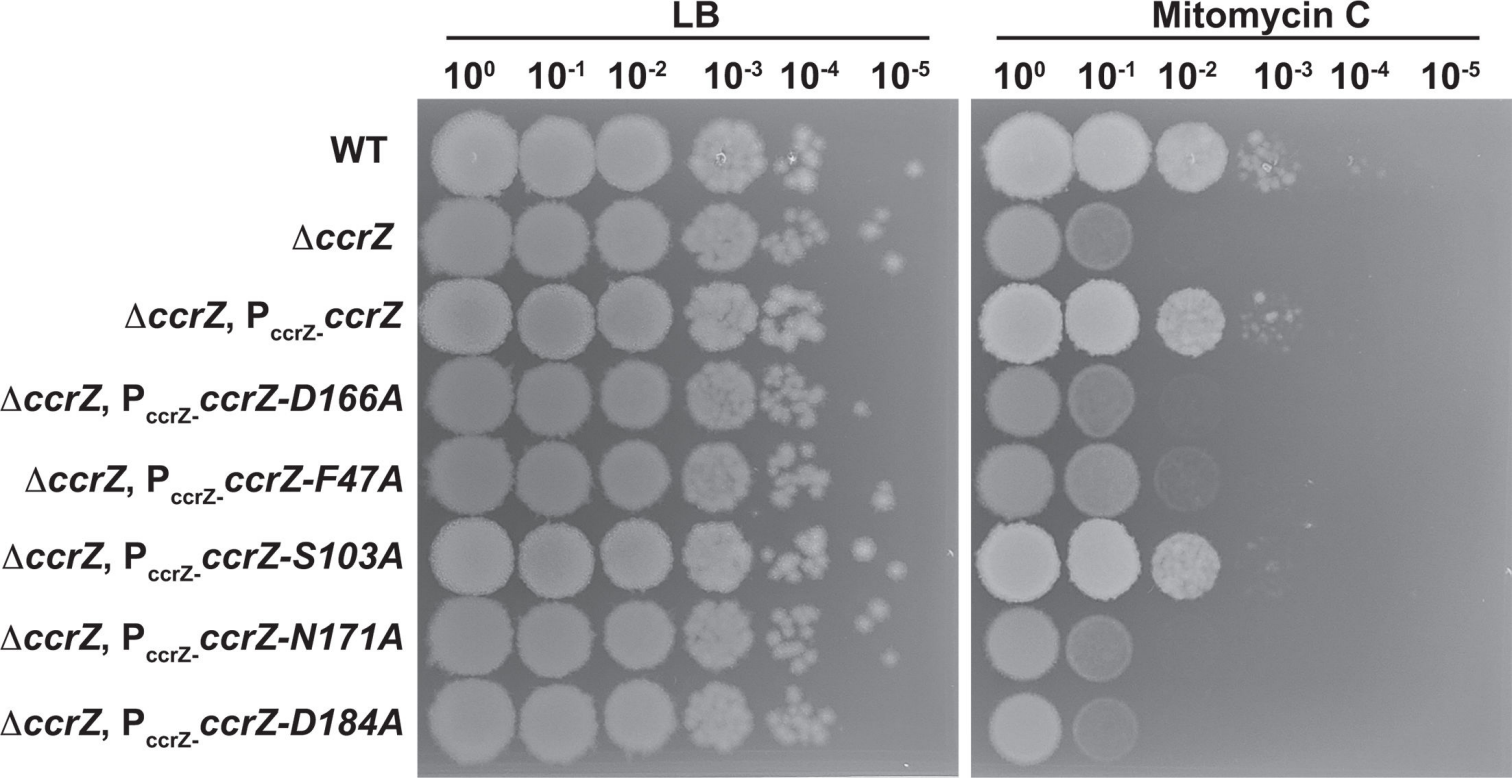
*ccrZ* alleles defective in replication initiation confer sensitivity to DNA damage. Shown is a spot titer assay with the indicated strains spotted onto LB or LB with 25 ng/mL mitomycin C. Each *ccrZ* allele is expressed from the *amyE* locus under the control of the native *ccrZ* promoter as described (35).

In our prior work, we showed that cells with a *yabA* null allele or certain *ccrZ* alleles conferred sensitivity to DNA damage (35). We show above that *ccrZ F47A*, *D166A, N171A*, and *D184A* yield similar DNA replication activity as Δ*ccrZ,* while *S103A* showed replication initiation activity similar to WT. We therefore tested each *ccrZ* allele for sensitivity to mitomycin C (MMC). We show that *F47A, D166A, N171A*, and *D184A* each confer sensitivity to MMC to the same extent as Δ*ccrZ,* while *S103A* and the complementing strain show WT growth in the presence of MMC, supporting previous results (Fig. 4) (35). We compared the MMC sensitivity of Δ*ccrZ* and *yabA* null cells to cells defective for DNA recombination to provide context for our results. We show that Δ*ccrZ* and *yabA* defective cells are sensitive to MMC but not to the same extent as cells lacking *recA* and *recN* (Fig. S3). Collectively, these experiments show that under-initiation and over-initiation of DNA replication are correlated with an increased sensitivity to genotoxic stress but do not render cells as susceptible as those defective in homologous recombination. We speculate that Δ*ccrZ* cells under-replicate, resulting in asynchronous initiation and inefficient homologous recombination, leading to growth interference in the presence of DNA damage.

### The DNA damage sensitivity of Δ*ccrZ* cells requires replication from *oriC*

**Fig 5:**
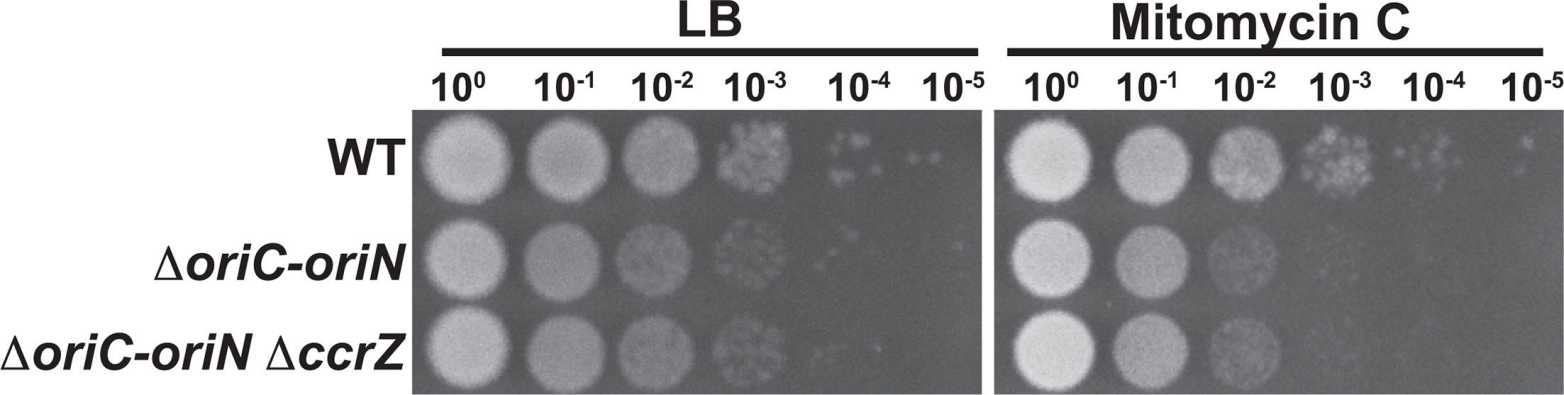
The DNA damage sensitivity of Δ*ccrZ* requires replication from *oriC*. Shown are spot titer plates of WT, Δ*oriC-oriN* and Δ*oriC-oriN* Δ*ccrZ* on LB agar plates or LB agar plates with 10 ng/mL mitomycin C after incubation at 30°C overnight.

Our demonstration here and in prior work (35) that Δ*ccrZ* results in sensitivity to DNA damage suggests that the sensitivity is caused by under-initiation. Another possibility is that *ccrZ* contributes to DNA repair in a process independent from its role in DNA replication initiation. To test if CcrZ contributes to DNA repair separate from its role in DNA replication initiation, we assayed the sensitivity of Δ*ccrZ* in cells that replicate from an alternative origin of replication (Fig. 5). It has been shown that the stimulatory effect of *ccrZ* on replication initiation requires the DnaA-dependent origin *oriC* (34). Therefore, we used cells with Δ*oriC* where chromosomal replication is under the control of a plasmid origin *oriN* and the replication initiation protein RepN (15, 37). We show that cells with *oriN* are exquisitely sensitive to DNA damage, which is expected given that replication from *oriN* causes replication forks to leave the origin in a way that increases DNA replication and transcription conflict (37), requiring us to use a low concentration of MMC for this experiment. We found no difference in the growth sensitivity of *oriN*-containing cells with or without *ccrZ* (Fig. 5). With our results, we conclude that the MMC sensitivity of Δ*ccrZ* requires replication from the DnaA-dependent origin *oriC,* providing strong support for the conclusion that under-replication in Δ*ccrZ* results in sensitivity to DNA damage.

### Over-initiation of DNA replication leads to replication fork stress

**Fig 6:**
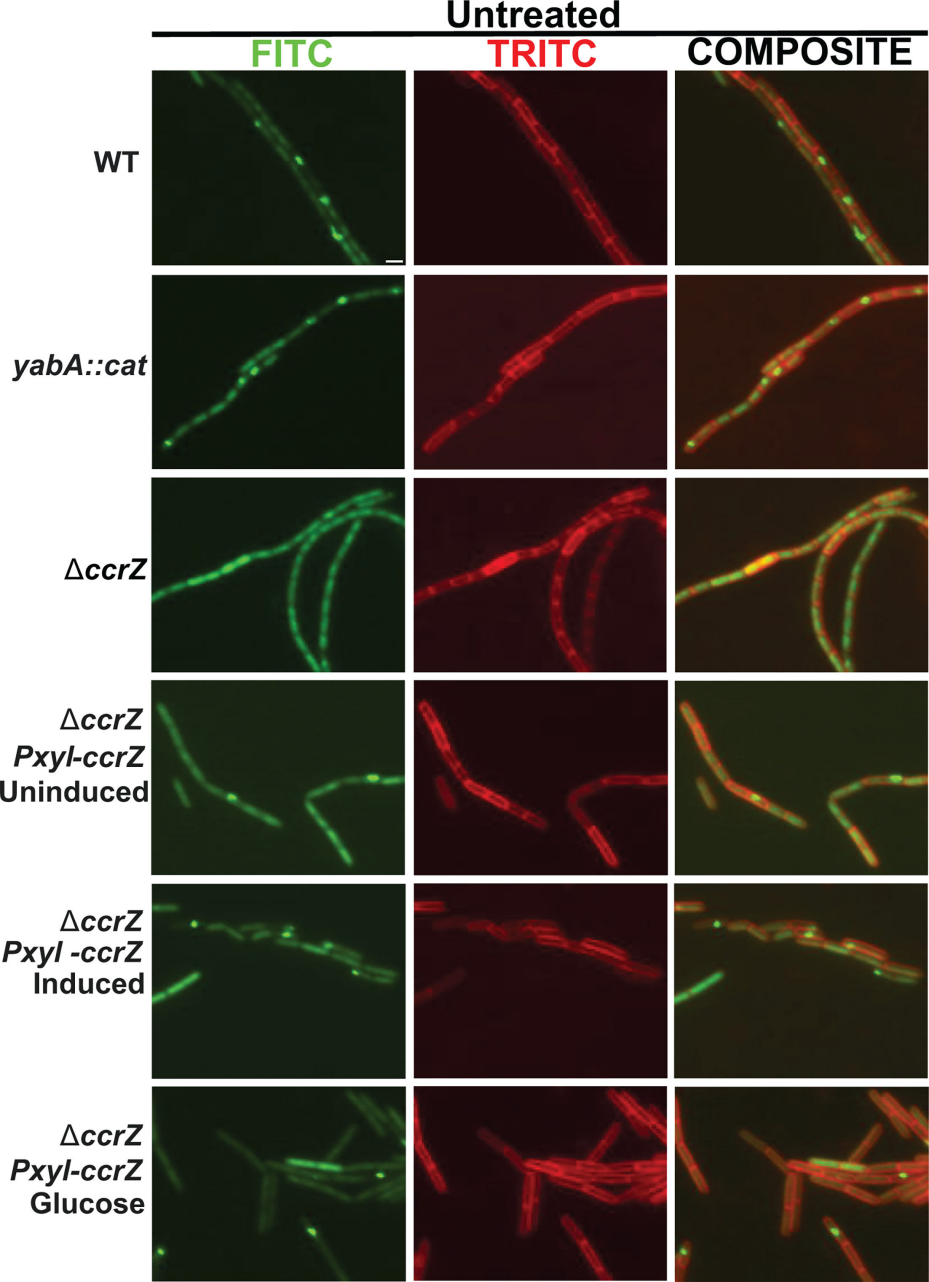
RecA-GFP foci are elevated in cells with increased DNA replication initiation. Shown are representative images of cells with the indicated genotypes. The leftmost column is RecA-GFP, followed by the middle column, membrane stained with FM 4-64, and the merge of the GFP and FM 4-64 channels (composite) to the right.

Prior work in *E. coli* showed that over-initiation of DNA replication through overproduction of DnaA or using the DnaAcos hyperactive variant resulted in replication fork stalling as determined by tracking replication fork progress using microarrays (19). When CcrZ was overproduced in *B. subtilis,* whole genome resequencing showed an increase in origin DNA, although the slope and marker frequency from the origin to the terminus provided no indication of replication fork stalling (35). Even though the slope of fork movement *in vivo* following CcrZ overexpression was inconsistent with fork stalling, we reasoned that cells could still experience replication fork stress without forks accumulating near the origin. Replication fork stress occurs when the fork encounters lesions, protein impediments, or other problems that generate excess ssDNA, leading to RecA binding (38, 39). Excess ssDNA bound by RecA leads to the formation of RecA-GFP foci at the replication fork in *B. subtilis* cells (38–40). Therefore, we used RecA-GFP as a proxy for replication fork stress in single *B. subtilis* cells following over-initiation of DNA replication by disrupting *yabA* or overproducing *ccrZ* (Fig. 6; Table 2). In WT cells, RecA-GFP foci form in ~12% (*n* = 2,550) of cells, supporting prior findings (38–40). Cells disrupted for *yabA* or overproducing *ccrZ* showed RecA-GFP foci in ~23.5 (*n* = 1,941) and ~37.4% (*n* = 891) of cells, respectively. In contrast, cultures of cells with Δ*ccrZ* only showed RecA-GFP foci in ~8.4% (*n* = 2,674) of cells, significantly lower than WT cells. These results show that elevated initiation of DNA replication leads to excess ssDNA that causes RecA-GFP to organize into foci, while under-initiation of DNA replication in Δ*ccrZ* results in less DNA and fewer RecA-GFP foci, suggesting that homologous recombination is reduced (see Discussion).

**TABLE 2:** Percentage of cells with RecA-GFP foci^*a*^

Strain name	Growth medium	Percentage of cells with foci (untreated)	Percentage of cells with foci (mitomycin C)
WT *recA-gfp*	S7_50_ glucose	12.3 ± 1.0 (*n* = 2,550)	43.5 ± 0.6 (*n* = 1,365)
*yabA::cat; recA-gfp*	S7_50_ glucose	23.5 ± 1.2 (*n* = 1,941)	78.5 ± 2.1 (*n* = 1,765)
Δ*ccrZ; amyE::P_xyl-_ccrZ; recA-gfp*	S7_50_ arabinose (uninduced)	3.9 + 0.5 (*n* = 1,333)	38.0 ± 7.0 (*n* = 987)
Δ*ccrZ; amyE::P_xyl-_ccrZ; recA-gfp*	S7_50_ arabinose (induced)	37.4 ± 5.4 (*n* = 891)	63.1 ± 4.9 (*n* = 773)
Δ*ccrZ; amyE::P_xyl-_ccrZ; recA-gfp*	S7_50_ glucose	9.3 ± 1.6 (*n* = 1,045)	17.9 ± 2.3 (*n* = 998)
Δ*ccrZ; recA-gfp*	S7_50_ glucose	8.4 ± 0.4 (*n* = 2,674)	33.0 ± 0.5 (*n* = 1,135)

^*a*^ Relationships between the untreated and treated conditions of each strain were found to be statistically significant by a Welch two-sample *t*-test. Standard error was calculated using the standard deviation and sample size. S7_50_ is the minimal medium used for the growth of each culture. *ccrZ* expression is induced with the addition of 1% xylose to S7_50_ with arabinose as a carbon source.

We asked if cells challenged with DNA damage showed elevated RecA-GFP foci following over-initiation of DNA replication. We reasoned that RecA-GFP foci formation would become exacerbated in damaged cells that also over-initiate DNA replication. We challenged WT cells with a concentration of MMC that would lead to elevated RecA-GFP foci without saturating the response (Fig. S4). For WT cells, we scored RecA-GFP foci in ~43.5% (*n* = 1,365) of cells following the addition of MMC. Cells deficient for *yabA* or overproducing *ccrZ* showed foci in ~78.5% (*n* = 1,765) and ~63.1% (*n* = 773) of cells, respectively, while cells with Δ*ccrZ* had RecA-GFP foci formation in ~33% of cells (*n* = 1,135), lower than that of WT (Fig. S5; Table 2). We wish to note that otherwise untreated cells overproducing CcrZ showed foci in ~37% of cells, while WT cells challenged with MMC formed foci in ~43% of cells. These results show that over-initiation results in a level of genome instability close to that of cells treated with an exogenous source of DNA damage. We conclude that over-initiation of DNA replication leads to changes in the formation of replication fork problems, which are further exacerbated by the presence of damaged template DNA. Furthermore, RecA-GFP foci are closely correlated with DNA replication initiation frequency. Cells that under-initiate replication have fewer foci than WT cells, and cells that over-initiate have more foci compared with WT. Our results are similar to the results found in *E. coli,* showing that the percentage of cells with RecA-GFP foci correlates with growth rate (41).

Since it has been shown in eukaryotes that over-initiation of replication leads to detrimental conflicts between DNA replication and transcription (27), we asked if the correlation between over-initiation and RecA-GFP foci formation was caused by replication forks encountering R-loops. In *B. subtilis,* RNase HIII is responsible for cleaving R-loops *in vitro* and *in vivo* (42, 43). Therefore, we overexpressed RNase HIII (*rnhC*) in cells with the *yabA* null allele and plated on MMC. If replication forks in over-initiating cells encounter R-loops leading to genome instability, we expect overexpression of RNase HIII to rescue or at least partially rescue the sensitivity of *yabA* null cells to DNA damage. We observed no rescue of *yabA* null cells when RNase HIII was overexpressed (Fig. S6). We did, however, find better survival of otherwise WT cells when *rnhC* was overproduced (Fig. S6), suggesting that R-loops are detrimental in WT cells that are also challenged with DNA damage. We also note that *yabA* null cells overproducing *ccrZ* cause an increased sensitivity to MMC, further demonstrating the connection between replication initiation frequency and sensitivity to DNA damage (Fig. S7). We conclude that R-loop formation does not underpin the DNA damage sensitivity observed during over-initiation.

## DISCUSSION

Here, we investigate the effect of under-initiation and over-initiation on genome integrity in *B. subtilis*. We examined *ccrZ* mutants that cause under-initiation and overproduced CcrZ and used a *yabA* null allele to cause over-initiation. We find that cells under-initiating DNA replication have few RecA-GFP foci, indicating that these cells incur less replication fork stress. We also find that cells under-initiating DNA replication are sensitive to DNA damage caused by MMC. This includes Δ*ccrZ, ccrZ (D166A), ccrZ (F47A), ccrZ (N171A*), and *ccrZ (D184A*) when these mutants are expressed from their native promoter. We suggest that under-initiating cells undergo asynchronous replication initiation, which leads to inefficient homologous recombination. Our finding also correlates with fewer RecA-GFP foci in cells that under-initiate, suggesting that forks experience fewer problems that require RecA, and recombination in general is reduced in cells that under-initiate replication. This could be caused in part by less homologous template available in Δ*ccrZ* cells due to under-initiation of replication.

In cells that over-initiate DNA replication, we observe a striking increase in both the sensitivity to DNA damage and RecA-GFP localization. Furthermore, another important finding from our work is that over-initiation of replication leads to nearly the same percentage of cells with RecA-GFP foci as WT cells directly damaged with the crosslinking agent MMC. This result suggests that over-initiation has a strong negative effect on genome integrity, resulting in substantial DNA damage. We conclude that an important outcome of regulating DNA replication initiation frequency is to maintain genome stability, as under- or over-initiation results in sensitivity to genotoxic stress.

CcrZ is a recently identified positive regulator of DNA replication initiation in *B. subtilis* (34, 35). CcrZ is a kinase, and it has been shown to interact with DnaA and DnaB through a bacterial two-hybrid assay (35). We measure *oriC/ter* ratios using *ccrZ* alleles under the control of the native promoter or following induced expression. We show that CcrZ(D166A), which is known to be defective for kinase activity, yields WT *oriC/ter* ratio when overexpressed and exhibits sensitivity to DNA damage. When *ccrZ(D166A*) is expressed from its own promoter, it shows an *oriC/terC* ratio identical to that of a Δ*ccrZ*. We suggest that the WT ratios measured in cells overproducing CcrZ(D166A) are due to weak stimulation of initiation through interaction with the *B. subtilis* replication initiation proteins and that the sensitivity to DNA damage likely results from asynchronous DNA replication initiation. The replication initiation stimulation observed with CcrZ(D166A) is far lower than when WT CcrZ is overproduced. With these data, we conclude that kinase activity from CcrZ provides the most important contribution to stimulating replication initiation activity, with interaction between CcrZ, DnaA, and DnaB providing some positive effect.

It has been clearly established in eukaryotes that under- and over-initiation of DNA replication leads to R-loop-mediated replication fork stress and DNA damage at fragile sites (for review, see reference 25). We overexpressed *B. subtilis* RNase HIII to test if R-loop-induced replication stress was occurring in cells undergoing over-initiation. We did not observe rescue, suggesting that R-loops either do not pose a major threat to genome stability in over-initiating cells or RNase HIII is inefficient for clearing R-loops encountered by replication forks under the conditions tested here. Because ectopic expression of RNase HIII yielded more survival to WT cells plated on DNA damage, we speculate that R-loops do threaten genome integrity when initiation frequency is normal. However, we further speculate that cells over-initiating DNA replication and contending with DNA damage are compromised to the point where R-loop removal by RNase HIII is unable to restore even partial growth.

We conclude that the proper regulation of DNA replication initiation is central to limiting replication fork stress and maintaining genome stability in bacterial cells that undergo multi-fork replication.

## MATERIALS AND METHODS

### Media and growth conditions

All strains and primers used in this study are described in Tables S1 and S2. All strains are derivatives of PY79 (44) and were constructed using standard procedures (45). Cultures for *oriC/ter* ratio generation were grown at 37°C in a water bath with shaking at 250 rpm. Lysogeny broth (LB medium: 10 g/L NaCl, 10 g/L tryptone, and 5 g/L yeast extract) or S7_50_ defined minimal medium with 2% glucose was used as described previously (35). In LB or S7_50_, xylose-inducible promoters were induced with 0.25% xylose. Antibiotics were used at the following final concentrations: spectinomycin (100 µg/mL), kanamycin (50 µg/mL), and chloramphenicol (5 µg/mL).

### Microscopy

Strains were grown on LB agar plates with the appropriate antibiotics and incubated overnight at 37°C. Cells were grown in S7_50_ minimal medium with 2% glucose or 1% arabinose as a carbon source. A volume of 12 mL of S7_50_ medium was added to a 125 mL Erlenmeyer flask and placed in a 30°C shaking water bath incubator for approximately 5–7 minutes. Then, 1.5 mL of the pre-warmed media was washed over the agar plate, and the optical density of the starting culture was measured between 0.080 and 0.1. The flask was wrapped tightly in aluminum foil, returned to the incubator, and grown at 30°C at 250 rpm until an OD_600_ value of 0.5–0.7 was reached. In order to assess the impact of DNA damage on the prevalence of RecA-GFP repair centers, cells were treated with the DNA crosslinking agent mitomycin C. A volume of 5 mL of the culture was transferred to a 50 mL Erlenmeyer flask, where 2.5 ng/mL of MMC was added. The flask was returned to the incubator and grown for 30 minutes prior to imaging.

After cultures reached an OD_600_ of 0.5–0.7 or following treatment with MMC, 300 µL of culture was combined with 1 µL (2 µg/mL) of FM4-64 membrane dye and gently agitated to incorporate the dye. Agarose (1%) was melted into 1× S7_50_ before being pipetted onto a standard 15-well glass slide. Cells were pipetted onto the agarose pads and allowed to settle for 7–10 minutes. Excess liquid was then aspirated from each well. A coverslip was set on top of the slide and adhered for approximately 2 minutes. Cells were imaged using an Olympus BX61 microscope under TRITC (exposure set between 200 and 500 ms) and FITC (exposure set between 400 and 800 ms) filters as described (38, 40, 46, 47). Shapiro-Wilk tests were performed to assess if microscopy data were normally distributed. The tests suggested that the data collected met the requirements for a normal distribution; thus, Welch’s *t*-tests were used to assess statistical significance between strains. In order to analyze data within a single strain and across conditions, dependent *t*-tests were used.

For image analysis of foci, fluorescent images were converted to 16-bit gray-scale images to measure area and relative fluorescence using FIJI. The ratio of relative fluorescence to area was calculated, and this ratio was used to determine if a region of interest qualified as a focus. A focus was defined as a region with a relative fluorescence per area ratio above 1.2.

### *oriC/ter* ratio generation via qPCR

In defined minimal medium or LB medium, cultures were started at an initial OD_600_ of 0.05 by diluting a plate wash into the respective medium. When cultures reached an OD_600_ between 0.2 and 0.4, they were back diluted to an OD_600_ of 0.05, grown to an OD_600_ of 0.2–0.4 again, and immediately arrested in ice-cold methanol in a 1:1 ratio of culture to methanol. The resulting mixture of methanol and culture was pelleted at 7,197 × *g* for 5 minutes. Genomic DNA was purified as described below. qPCR was used to generate origin (*oriC*) to terminus (*ter*) ratios, quantifying the relative amounts of each via the Pfaffl method as described (16, 48). Control strain *dnaB134* (TTR1) was grown identically to other samples, followed by an incubation at the non-permissive temperature of 45°C for two additional hours in a still bath. This allows completion of ongoing rounds of DNA replication but prevents the initiation of new rounds of replication, resulting in a 1:1 ratio of origin to terminus DNA. *oriC/ter* ratios were generated using average control values across all qPCR runs and normalized to controls in individual qPCR runs. qPCR was performed with 20 µL reactions containing 10 µL SsoAdvanced Universal SYBR Green Supermix, 250 nM of each primer, and 4 ng of gDNA. StepOnePlus Real-Time PCR System was used for all qPCR runs. Primers targeting the origin region were oTTR1 (5′-TTGCCGCAGATTGAAGAG-3′) and oTTR2 (5′-AGGTGGACACTGCAAATAC-3′), while primers targeting the terminus region were oTTR3 (5′-CGCGCTGACTCTGATATTATG-3′) and oTTR4 (5′-CAAAGAGGAGCTGCTGTAAC-3′). Primers are also listed in Table S2 and were used in reference 16.

### Genomic DNA purification

Cell pellets from 25 mL cultures were resuspended in 200 µL of lysis buffer (50 mM Tris-HCl [pH 8], 10 mM EDTA [pH 8], 1% Triton X-100, 0.5 mg/mL RNase A, and 5 mg/mL lysozyme) followed by incubation for 30 minutes at 37°C. Next, 40 µL of 10% SDS and Proteinase K were added and mixed by pipette. Samples were subsequently incubated at 55°C for 55 minutes. A volume of 600 µL PB buffer (5 M guanidine-HCl and 30% [vol/vol] isopropanol) was added, gently mixed, and the sample was applied to the EconoSpin spin column for DNA and centrifuged for 1 min at 12,000 × *g*. Five hundred microliters of PB was used to wash the column and centrifuged at the same speed and time as before. A volume of 500 µL of PE (10 mM Tris-HCl [pH 7.5] and 80% [vol/vol] ethanol) was added for a final wash, centrifuged at 12,000 ×  *g* for 1 minute, followed by an additional centrifugation of the dry column for a minute at 12,000 × *g*. Tris-HCl (10 mM, pH 8) was used to elute the DNA, centrifuging at 14,000 *× g* for 1 minute. The eluent was reloaded onto the column and centrifuged at 14,000 *× g* for 1 minute.

## Data Availability

The raw data for the qPCR experiments underlying the data in Table 1, Fig. 1 to 3, and Fig. S1 are deposited at https://figshare.com/browse. The updated summary files are located at the following link: https://doi.org/10.6084/m9.figshare.28858673.v1.

## SUPPLEMENTAL MATERIAL

Supplemental figures and tables jb.00175-25-s0001.pdf Fig. S1 to S7, and Tables S1 and S2.

ASM does not own the copyrights to Supplemental Material that may be linked to, or accessed through, an article. The authors have granted ASM a non-exclusive, world-wide license to publish the Supplemental Material files. Please contact the corresponding author directly for reuse.
