# Supplementary material for: DNA replication initiation timing is important for maintaining genome integrity
Source: J Bacteriol. 2025 Jul 21;207(8):e00175-25. doi: 10.1128/jb.00175-25 (PMC12369353; doi:10.1128/jb.00175-25)
Supplement: Supplemental figures and tables — Fig. S1 to S7, and Tables S1 and S2. [file jb.00175-25-s0001.pdf]

SUPPLEMENTAL INFORMATION

DNA replication initiation timing is important for maintaining genome integrity

Tristan T. Reed[1], Abigail H. Kendal[1], Katherine J. Wozniak[1,2] and Lyle A. Simmons[1*]

[1]Department of Molecular, Cellular, and Developmental Biology, University of Michigan, Ann Arbor, MI 48109

[2]Present address: Department of Molecular Virology and Microbiology, Baylor College of Medicine, Houston, TX 77030

*LAS: Department of Molecular, Cellular, and Developmental Biology, University of Michigan, Ann Arbor, Michigan 48109-1055, United States. Phone: (734) 763-7142, Fax: (734) 647-0884 E-mail: lasimm@umich.edu

Short title: DNA replication initiation impacts genome integrity

Keywords: CcrZ, *Bacillus subtilis,* DnaA, DNA replication, RecA

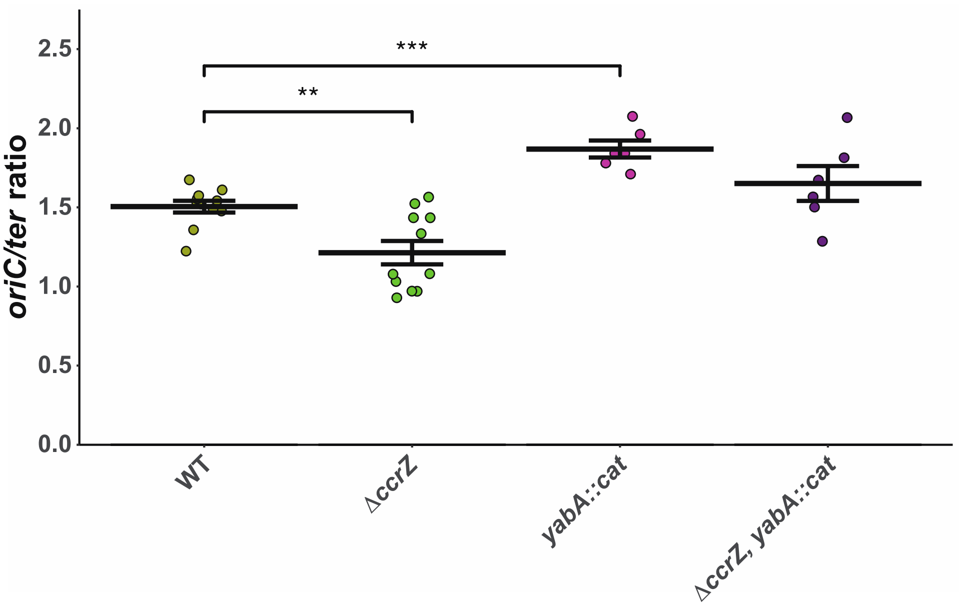

Figure S1. Relative *oriC/ter* ratios of cultures grown in $S7_{50}$ minimal medium with 1% arabinose.

Shown are qPCR-generated *oriC/ter* ratios for *B. subtilis* strains grown in $S7_{50}$ minimal medium with 1% arabinose at 37°C. Data points are plotted along with their means repressented by the horizontal bar and error bars represent the standard error of the mean for at least six biological replicates. Statistically significant increase or decrease in ratios assessed by two-tailed t-test in relation to WT, *p < 0.05, **p < 0.01, ***p < 0.001, ****p < 0.0001.

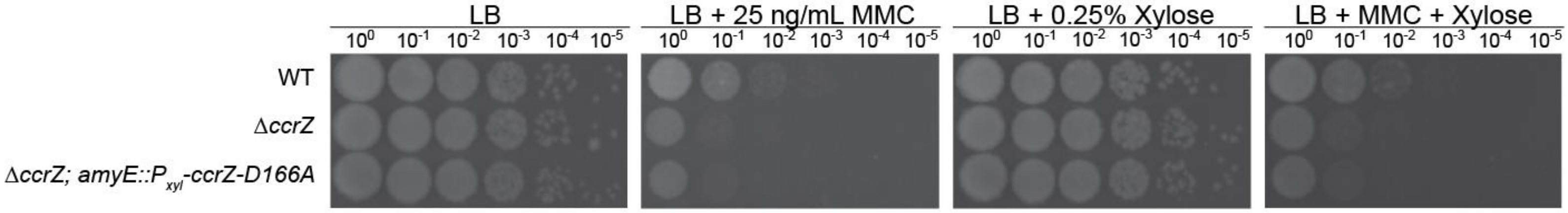

Figure S2. Ectopic expression of *ccrZ(D166A)* causes sensitivity to DNA damage. Shown are spot plate assays with WT, Δ*ccrZ* and xylose induced expression of *ccrZ(D166A)*. Strains were serial diluted and plated on LB medium with mitomycin C (25 ng/mL) and xylose (0.25%).

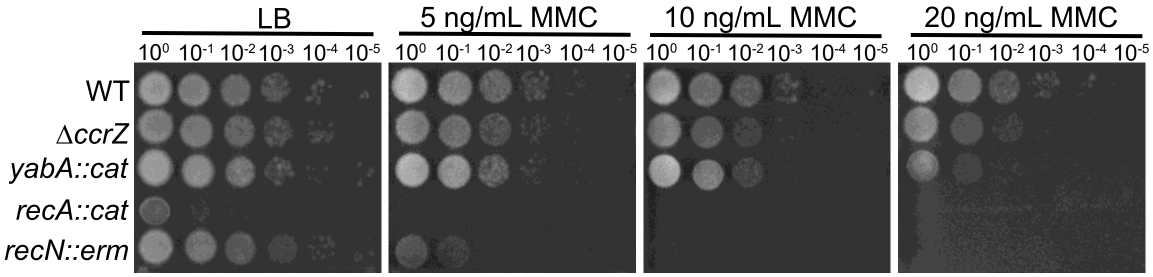

Figure S3. Loss of *ccrZ* and *yabA* causes sensitivity to DNA damage.
Shown are spot titer plates with cells lacking *ccrZ*, *yabA*, *recA* and *recN* on LB agar or LB agar plates with the indicated concentration of MMC.

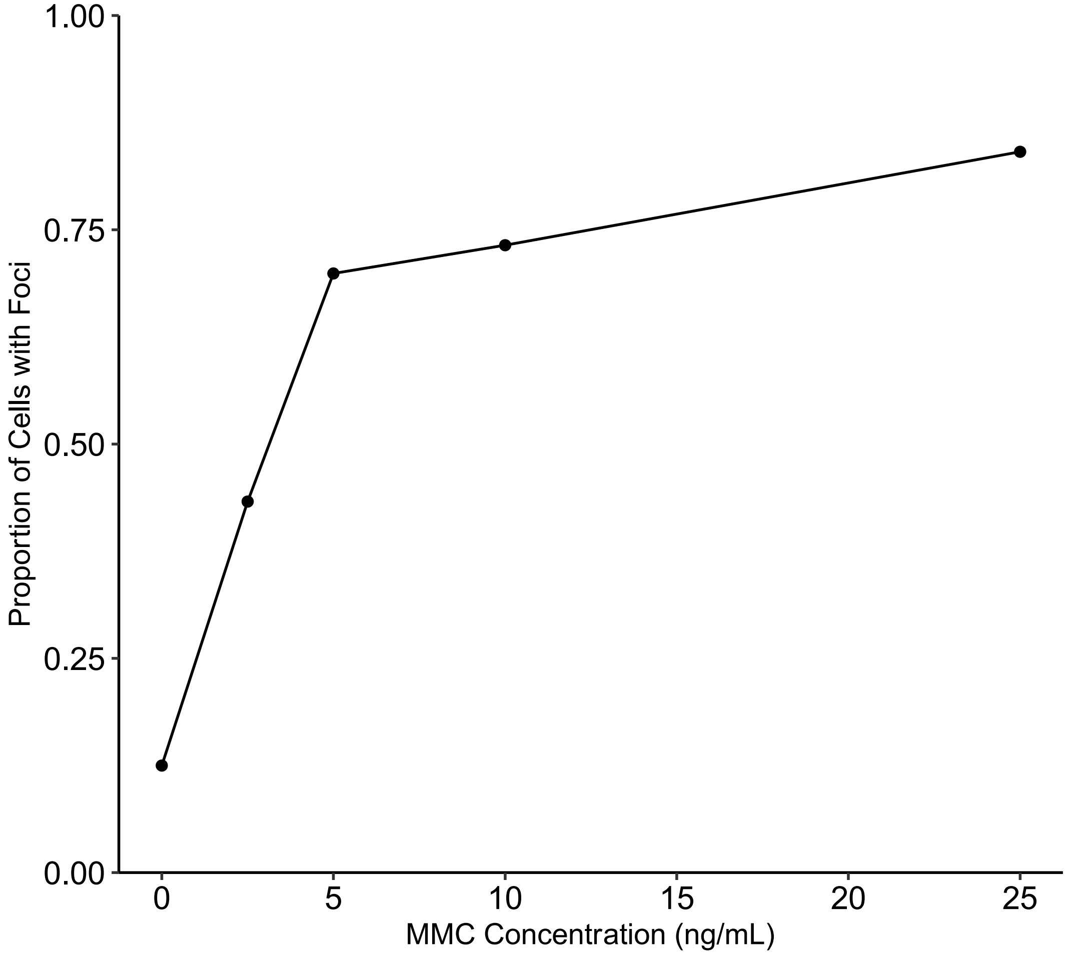

Figure S4. Organization of RecA-GFP into foci in response to mitomycin C concentration. The proportion of cells with RecA-GFP foci were determined in response to the concentration of mitomycin C (MMC) added to the culture medium. Cells were challenged with the indicated concentration of MMC for 30 minutes prior to imaging. The percent of cells with foci and the number of cells scored (n) are as follows: untreated (12.5%, n=594); 2.5 ng/mL (43.3%, n=531); 5 ng/mL (69.9%, n=596) 10 ng/mL (73.2%, n=504); 25 ng/mL (84.1%, n=584). Based on this data 2.5 ng/mL was chosen as the appropriate concentration of MMC to test for RecA-GFP foci in cells over and under-initiating DNA replication.

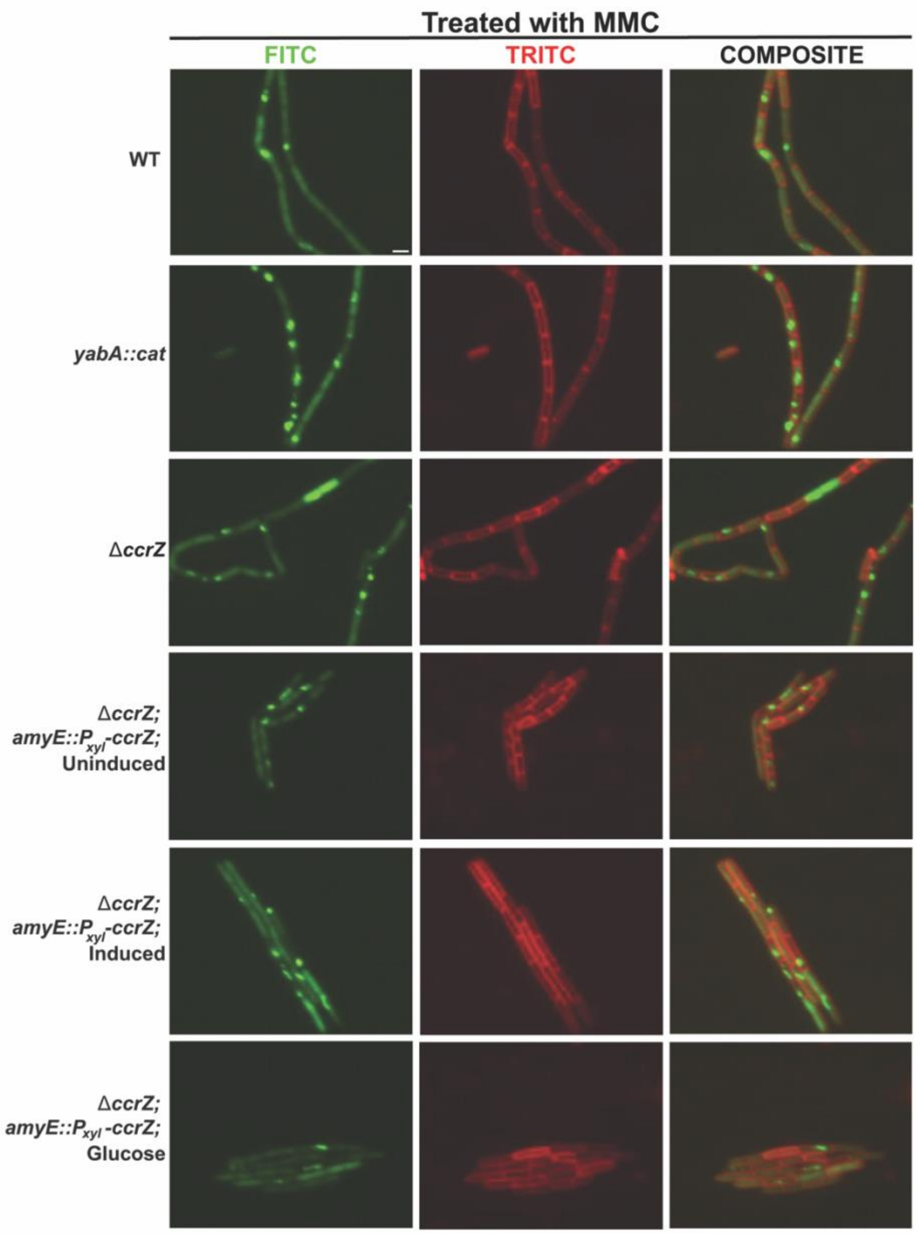

Figure S5. Cells during over-initiation of DNA replication have more RecA-GFP foci.

Shown are representative micrographs of cells with the indicated genotypes grown normally in $S7_{50}$ minimal medium and challenged with 2.5 ng/ml mitomycin C for 30 minutes prior to imaging. The left most column is RecA-GFP, followed by the membrane stained with FM 4-64 followed by the merge of the GFP and FM 4-64 channels (composite).

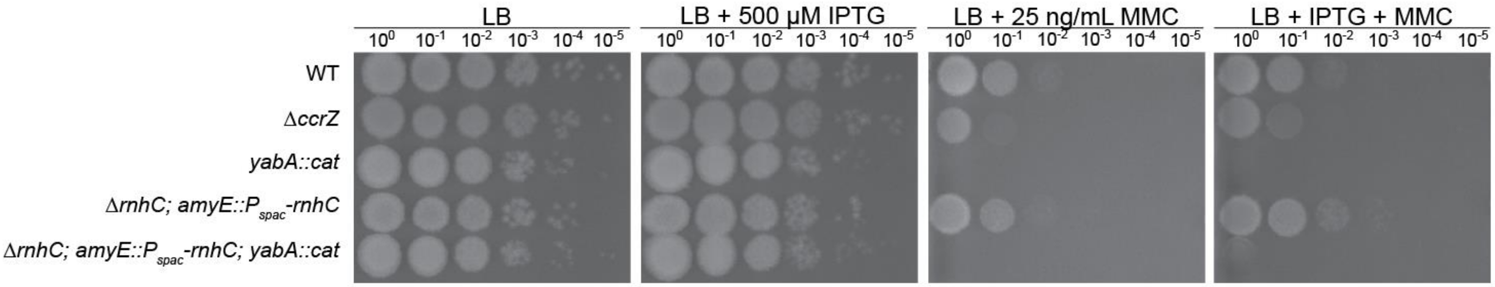

Figure S6. Overexpression of RNase HIII does not alleviate growth interference caused by over-initiation of DNA replication. Shown are spot titer plates of the indicated strains on LB or LB with 25 ng/ml mitomycin C. Expression of *rnhC* (RNase HIII) is driven by the IPTG regulated P_{spac} promoter.

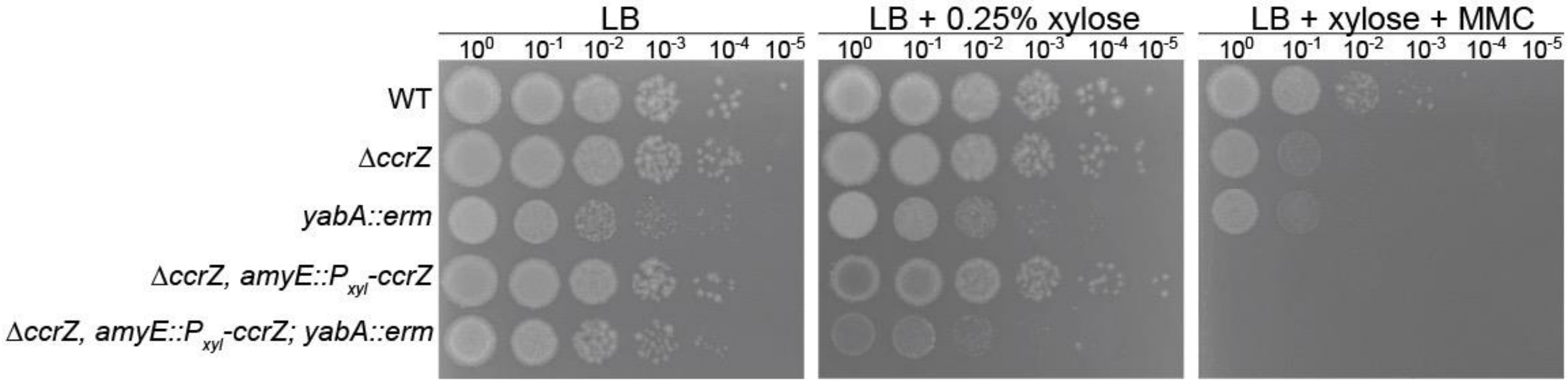

Figure S7. Expression of *ccrZ* in *yabA* defective cells results in growth interference and sensitivity to DNA damage. Shown are spot titers on the indicated medium with increasing 10-fold serial dilutions. Expression of CcrZ is driven by xylose using the P_{xyl} promoter with MMC added to 25 ng/ml.

Table S1. Strains used in this study.

Strain	Relevant Genotype	Source
PY79	WT, prototroph SPβ^{o}	(1)
TTR1	*dnaB134(ts)*; *zhb83::Tn917* (Cm^{r})	(2)
TTR4	Δ*ccrZ*	(3)
TTR7	Δ*ccrZ*, *amyE*::P_{xyl}-*ccrZ* (Cm^{r})	(3)
TTR10	ΔccrZ, *amyE*::P_{xyl}-*ccrZ*-D166A (Cm^{r})	(3)
TTR19	*yabA::cat* (Cm^{r})	(4)
TTR22	Δ*ccrZ, yabA::cat* (Cm^{r})	(3)
TTR28	Δ*ccrZ*, *amyE*::P_{ccrZ}-*ccrZ-D166A* (Cm^{r})	(3)
TTR31	Δ*ccrZ*, *amyE*::P_{ccrZ}-*ccrZ-F47A* (Cm^{r})	(3)
TTR34	Δ*ccrZ*, *amyE*::P_{ccrZ}-*ccrZ-S103A* (Cm^{r})	(3)
TTR37	Δ*ccrZ*, *amyE*::P_{ccrZ}-*ccrZ-N171A* (Cm^{r})	(3)
TTR40	Δ*ccrZ*, *amyE*::P_{ccrZ}-*ccrZ-D184A* (Cm^{r})	(3)
TTR43	Δ*ccrZ,amyE::*P_{ccrZ}*-ccrZ* (Cm^{r})	(3)
TTR46	Δ*ccrZ, amyE::*P_{ccrZ}*-ccrZ-D166A* (Cm^{r})	(3)
AHK1	*recA::recA-gfp* (Spc^{r})	(5)
AHK6	*yabA::cat, recA::recA-gfp* (Spc^{r})	This work
AHK18	Δ*ccrZ, recA::recA-gfp* (Spc^{r})	KJW868
AHK16	Δ*ccrZ, amyE*::P_{xyl}-*ccrZ* (Cm^{r})*, recA::recA-gfp* (Spc^{r})	KJW870
AHK20	Δ*rnhC*, amyE::P_{spac}-*rnhC*, *yabA::cat*	This work
AHK26	Δ*ccrZ, amyE*::P_{xyl}-*ccrZ* (Cm^{r}), *yabA::erm*	This work
AHK47	*recA*::*cat*	(6)
AHK49	*recN*::*erm*	PEB85
LAS927	(Δ*oriC*-L-*dnaN*) Kan^{r}-*oriN-repN*-P_{pen}2027*dnaN*	Grossman Lab
LAS929	Δ*ccrZ,* (Δ*oriC*-L-*dnaN*) Kan^{r}-*oriN*-repN-P_{pen}2027*dnaN*	

Table S2. Primers used in this study.

Primer	Sequence (5′-3′)
oTTR1	TTGCCGCAGATTGAAGAG
oTTR2	AGGTGGACACTGCAAATAC
oTTR3	CGCGCTGACTCTGATATTATG
oTTR4	CAAAGAGGAGCTGCTGTAAC

REFERENCES

1. Youngman P, Perkins JB, Losick R. 1984. Construction of a cloning site near one end of Tn917 into which foreign DNA may be inserted without affecting transposition in Bacillus subtilis or expression of the transposon-borne erm gene. Plasmid 12:1-9.
2. Walsh BW, Bolz SA, Wessel SR, Schroeder JW, Keck JL, Simmons LA. 2014. RecD2 helicase limits replication fork stress in Bacillus subtilis. J Bacteriol 196:1359-68.
3. Wozniak KJ, Burby PE, Nandakumar J, Simmons LA. 2022. Structure and kinase activity of bacterial cell cycle regulator CcrZ. PLoS Genet 18:e1010196.
4. Goranov AI, Breier AM, Merrikh H, Grossman AD. 2009. YabA of Bacillus subtilis controls DnaA-mediated replication initiation but not the transcriptional response to replication stress. Mol Microbiol 74:454-66.
5. Simmons LA, Grossman AD, Walker GC. 2007. Replication is required for the RecA localization response to DNA damage in Bacillus subtilis. Proc Natl Acad Sci U S A 104:1360-5.
6. McLean EK, Lenhart JS, Simmons LA. 2021. RecA Is Required for the Assembly of RecN into DNA Repair Complexes on the Nucleoid. J Bacteriol 203:e0024021.
